# INVEST study warns on too-low BP in diabetic patients with CAD

**Published:** 2010-04

**Authors:** J Aalbers

**Affiliations:** Special Assignments Editor

## Introduction

The INVEST (INternational VErapamil SR-Trandolapril) study, a very large international study of hypertensive patients, has shown that patients with type 2 diabetes and coronary artery disease do not benefit from tightening systolic blood pressure levels to below 130 mmHg, and this may in some cases be harmful.

INVEST, initiated in the mid-nineties, continues to contribute to the improved management of patients with CAD and this latest evaluation of the 6 400 patients with diabetes and CAD included in the INVEST cohort is the first study to critically evaluate the effects of systolic blood pressure lowering in patients with both diabetes and documented CAD.

These results are likely to require guideline committees, including the local SEMDSA’s guideline committee, to include a warning that blood pressure need not be driven below 130 mmHg in diabetic patients as this does not result in any further cardiovascular benefit.

This finding is particularly pertinent to drug selection and the use of verapamil SR/trandolapril, which was successful with more than 70% of INVEST patients reaching the target blood pressure of less than 140/90 mmHg. The verapamil SR/ trandolapril therapy group also experienced significantly fewer cases of newonset diabetes than those patients treated with atenolol/hydrochlorothiazide.

‘Current guidelines suggest “lower is better” with regard to blood pressure’, said Rhonda M Cooper-DeHoff, PharmD, MS, and associate professor of pharmacy and medicine at the University of Florida, Gainesville. ‘The INVEST data suggest that in patients with both diabetes and coronary artery disease, there is a blood pressure threshold below which cardiovascular risk increases’.

For the study, INVEST randomly assigned 6 400 patients with diabetes and CAD to blood pressure-lowering therapy based on either a calcium channel blocker or a beta-blocker, plus an angiotensin converting enzyme (ACE) inhibitor and/ or a thiazide diuretic. The target was a blood pressure of < 130/< 85 mmHg.

For the analysis, patients were categorised according to the degree of blood pressure control actually achieved. Patients with a systolic blood pressure of 140 mmHg or higher, almost onethird of patients, were classified as ‘not controlled’. Those with a systolic blood pressure below 130 mmHg were classified as ‘tight control’ and those with a systolic blood pressure in between (≥ 130 mmHg, but < 140 mmHg) were classified as ‘usual control’.

During a follow-up period equivalent to more than 16 893 patient-years, researchers found that patients in the notcontrolled group had nearly a 50% higher combined risk of death, heart attack or stroke when compared with the usual-care group. However, those in the tight-control group had a similar risk to those in the usual-control group.

Further analysis showed that lowering systolic blood pressure below 130 mmHg significantly increased the risk of allcause death when compared to usual care, an increase that became apparent about 30 months into the study and persisted for an additional five years of follow up. When researchers then analysed blood pressure in 5-mmHg increments in the tight-control group, they discovered that a systolic blood pressure below 115 mmHg was associated with increased mortality.

‘Diabetic patients with CAD in whom blood pressure is not controlled have increased risk for unfavourable cardiovascular outcomes, so the message to lower systolic blood pressure below 140 mmHg is still important’, Cooper-DeHoff said. ‘However, it is not necessary to lower systolic blood pressure below 130 mmHg to reduce that risk. Most importantly, reducing systolic blood pressure below 115 mmHg may be associated with increased mortality.’

